# Expression and Localization of *Kcne2* in the Vertebrate Retina

**DOI:** 10.1167/iovs.61.3.33

**Published:** 2020-03-19

**Authors:** Moritz Lindner, Michael J. Gilhooley, Teele Palumaa, A. Jennifer Morton, Steven Hughes, Mark W. Hankins

**Affiliations:** 1 Nuffield Laboratory of Ophthalmology, Sleep and Circadian Neuroscience Institute, Nuffield Department of Clinical Neurosciences, University of Oxford, Oxford, United Kingdom; 2 Oxford Eye Hospital, Oxford University Hospitals NHS Foundation Trust, Oxford, United Kingdom; 3 Department of Neurophysiology, Institute of Physiology and Pathophysiology, Philipps University, Marburg, Germany; 4 Moorfields Eye Hospital, London, United Kingdom; 5 Department of Physiology, Development, and Neuroscience, University of Cambridge, Cambridge, United Kingdom

**Keywords:** *Kcne2*, MinK-related peptide 1, Mirp1, ion channels, photoreceptors, bipolar cells, ribbon-synapse

## Abstract

**Purpose:**

To characterize the retinal expression and localization of Kcne2, an ancillary (β) ion-channel subunit with an important role in fine-tuning cellular excitability.

**Methods:**

We analyzed available single-cell transcriptome data from tens of thousands of murine retinal cells for cell-type-specific expression of *Kcne2* using state-of-the-art bioinformatics techniques. This evidence at the transcriptome level was complemented with a comprehensive immunohistochemical characterization of mouse retina (C57BL/6, ages 8–12 weeks) employing co-labeling techniques and cell-type-specific antibody markers. We furthermore examined how conserved the Kcne2 localization pattern in the retina was across species by performing immunostaining on zebrafish, cowbird, sheep, mice, and macaque.

**Results:**

*Kcne2* is distinctly expressed in cone photoreceptors and rod bipolar cells. At a subcellular level, the bulk of Kcne2 immunoreactivity can be observed in the outer plexiform layer. Here, it localizes into cone pedicles and likely the postsynaptic membrane of the rod bipolar cells. Thus, the vast majority of Kcne2 immunoreactivity is observed in a thin band in the outer plexiform layer. In addition to this, faint Kcne2 immunoreactivity can also be observed in cone inner segments and the somata of a small subset of cone ON bipolar cells. Strikingly, the localization of Kcne2 in the outer plexiform layer was preserved among all of the species studied, spanning at least 300 million years of evolution of the vertebrate kingdom.

**Conclusions:**

The data we present here suggest an important and specific role for Kcne2 in the highly specialized photoreceptor-bipolar cell synapse.

K*cne2*, originally termed MinK-related peptide 1, codes for an ancillary (β) ion-channel subunit that governs gating, subunit composition, and directed trafficking for a multitude of ion channels.^1^^,^^2^ Beyond the field of visual neurosciences, *Kcne2* has received major attention, as mutations in this gene have been associated with a severe form of inherited cardiac arrhythmias, the long-QT 6 syndrome (LQT6).^1^^,^^3^ Interestingly, despite the confirmed high relevance of *Kcne2* for the heart and several other organ systems,^1^ a detailed analysis of *Kcne2* in the retina on a structural or functional level is still missing.

Structural evidence on *Kcne2* has so far been limited to a study on bovine retina that reported *Kcne2* expression in the outer plexiform layer (OPL),^4^ the level where sensory photoreceptors synapse with their second-order neurons, the bipolar cells. More recently, a seminal work in the field of single-cell RNA sequencing provided in-depth transcriptomic data for individual cell types of the retina.^5^ In that study, *Kcne2* was implicated as a marker gene for cone photoreceptors in mice. Photoreceptors, including their synapses, are highly specialized: A complex and incompletely understood cellular machinery is in place to fine-tune the ion-channel composition of various subcellular structures, including the presynaptic terminal.^6^ Deleterious alterations of the electrical properties of photoreceptors or the photoreceptor–bipolar cell synapse are associated with retinal degeneration and congenital stationary night blindness. Interestingly, such impairment does not necessarily have to arise from direct (e.g., genetic or toxic) impairment of ion channels; for example, it has been shown that functional defects of interaction partners such as RS1 (OMIM 300839) or nyctalopin (OMIM 300278) can cause retinal degeneration and congenital stationary night blindness, respectively.^7^^–^^9^

Given that the limited available evidence^4^^,^^5^ suggests a highly distinct expression pattern of *Kcne2* in the retina, a refined analysis of the expression and localization of *Kcne2* is important. In the present study, we combined a bioinformatic approach analyzing single-cell RNA sequencing with in-depth immunohistochemistry from mice and a spectrum of representative members of the vertebrate kingdom in order to characterize the expression patterns and localization of Kcne2 in the retina. We find that Kcne2 is distinctly expressed in cone photoreceptors and rod bipolar cells and in both cell types predominantly localizes to the photoreceptor–bipolar cell synapse in all species studied.

These data add a new perspective onto the protein machinery that is in place at the photoreceptor–bipolar cell synapse to potentially shape its electrical activity. They indicate that *Kcne2* is likely to perform an important physiological role in vision.

## Methods

### Animals

All procedures were conducted in accordance with the UK Home Office Animals (Scientific Procedures) Act 1986 and the ARVO Statement for the Use of Animals in Ophthalmic and Vision Research. Mice (*Mus musculus*) used in this study were obtained from an internal breeding program at the University of Oxford (C57BL/6JOlaHsdOxuni). They were 8 to 12 weeks old and were housed under a 12-hour light/dark cycle at 21°C; diet and water were available ad libitum. Tissue was collected at Zeitgeber time (hours after light onset) 3 to 5 following cervical dislocation. Monkey (*Macaca mulatta*; 7 years of age) tissue was obtained from the Oxford primate tissue sharing initiative. Tissue from sheep (*Ovis aries*), Argentinian cowbird (*Molothrus rufoaxillaris*), and zebrafish (*Danio rerio*) was originally collected as part of previous and ongoing studies (Hughes S and Hankins MW unpublished).^10^ Cowbird retina was obtained via Alex Kacelnik, University of Oxford, and Juan Reboreda, University of Buenos Aires.

### Bioinformatics

For analysis of *Kcne2* expression at the mRNA level, we analyzed the aligned single-cell RNA-sequencing dataset as published in Macosko et al.^5^ that contained 44,808 retinal cells from p14 mice (downloaded from the Gene Expression Omnibus server, www.ncbi.nlm.nih.gov/geo/; accession number GSE63472). Processing and analysis of the dataset were performed using R 3.5.1^11^ and Seurat 2.3.4 (Satija Lab, New York, NY, USA).^12^ Data were log-normalized, centered, and scaled. Assignment of individual cells into clusters (i.e., cell types) was adopted from the original publication,^5^ utilizing the assignment matrix as published by the authors (www.mccarrolllab.org/dropseq/). To identify the subtypes represented by the identified bipolar cell clusters, the top five marker genes for the respective cluster were analyzed. Phylogenetic analyses of *Kcne2* expression in the retina were carried out using TimeTree^13^ and visualized using R and the rotl, ggtree, and phylopic (http://phylopic.org/) packages.^14^^,^^15^ Orthologs of *Kcne2* in the analyzed species were identified using OrthoDB, and sequences of all orthologs were aligned to the sequence of the immunogenic peptide used to raise the *Kcne2* antibody utilized here (see below).^16^^,^^17^

### Cell Culture and Transfection

Chinese hamster ovary cells were grown in Minimum Essential Medium α supplemented with 10% fetal calf serum and 1% penicillin/streptomycin (all Invitrogen GmbH, Darmstadt, Germany) in a humidified atmosphere at 5% CO_2_ and 37°C and passaged every 3 to 4 days as previously described.^18^ For experiments, cells were cultured on polylysine-coated glass coverslips and transfected 1 to 2 days after seeding using GeneJuice transfection reagent (Merck KGaA, Darmstadt, Germany) according to the manufacturer's instructions. *Kcne2* (NM_134110.3) subcloned into the pcDNA3.1 mammalian expression vector was obtained from Genscript (Piscataway, NJ, USA).

### Immunohistochemistry

For histological analysis, tissue was collected and processed according to in-house standard procedures.^19^ Eyes were punctured with a fine-gauge needle, fixed in 4% methanol-free paraformaldehyde (Thermo Fisher Scientific, Waltham, MA, USA) in PBS for 24 hours, then transferred to 30% (w/v) sucrose in H_2_O and stored at 4°C for >48 hours. For primate and sheep eyes, the anterior segment was subsequently removed. For preparation of retinal cryosections eyes were embedded into optimal cutting temperature medium (VWR International, Lutterworth, UK) and stored at –80°C until further processing. Then, 18-µm tissue sections were prepared using Cryotome FSE (Thermo Fisher Scientific). Cell monolayers were fixed in 4% methanol-free paraformaldehyde (Thermo Fisher Scientific) in PBS for 10 minutes and subsequently washed and stored until further use. Fluorescent immunolabeling was performed using standard techniques as previously described.^19^ Briefly, retinal sections were permeabilized in PBS with 0.2% Triton X-100 (Sigma-Aldrich, St. Louis, MO, USA) and blocked in PBS with 10% normal donkey serum. Sections were then incubated with primary antibodies for 24 hours at 4°C. A list of all primary antibodies employed in this study is given in the Table. Subsequent incubation with secondary antibodies was performed for 2 hours at room temperature (21°C). Secondary antibodies raised in donkey and labeled with either Alexa Fluor 488 or Alexa Fluor 568 fluorophores (Life Technologies, Carlsbad, CA, USA) were utilized at 1:1000. In some experiments, Alexa Fluor conjugated peanut agglutinin was employed to label cone photoreceptors. Incubation was performed together with the secondary antibodies at a dilution of 1:50.

**Table.** Primary Antibodies Used in This Study

Target	Host	Antibody	Dilution
Kcne2 (residues 88–107 in rat sequence, P63161)	Rabbit	Alomone Labs APC-054	1:800–1:1600
Snap25	Rabbit	Abcam ab41455	1:1000
PKCα	Rabbit	Abcam ab32376	1:1000
PKCα	Mouse	Santa Cruz Biotechnology sc-8393	1:500
Chx10	Sheep	Abcam ab16141	1:500
Cone arrestin	Rabbit	Merck AB15282	1:500
GlyT-1	Goat	Thermo Fisher Scientific AB1770	1:1000
GABA	Mouse	Sigma-Aldrich GB-69	1:2500
ChAT	Goat	Merck AB144P	1:1000
Peanut agglutinin	—	Thermo Fisher Scientific L21409 and L32460	1:50

Where stated, sections were also co-stained with various primary antibodies raised in the same host species. In these cases, labeling was performed sequentially; between the individual rounds of staining, sections were blocked in 10% serum of the species of the primary antibody and incubated for 60 minutes with unconjugated Fab antibody fragments raised against the host species of the primary antibodies (AffiniPure donkey anti-rabbit IgG [H+L], 711-007-003;Jackson ImmunoResearch Laboratories, West Grove, PA, USA). All antibodies were diluted in PBS containing 2.5% normal donkey serum and 0.2% Triton X-100. Nuclear counterstaining was performed with 4′,6-diamidino-2-phenylindol (0.5 µg/ml in PBS) for 10 minutes. Sections were mounted with Prolong Gold Antifade media (Life Technologies).

### Image Acquisition and Analysis

Fluorescence images were collected using an inverted LSM 710 laser scanning confocal microscope (Carl Zeiss Meditec, Oberkochen, Germany) or an inverted Fluoview FV1000 (Olympus, Tokyo, Japan). Individual channels were collected sequentially. Laser lines for excitation were 405 nm, 488 nm, and either 561 nm (LSM 710) or 559 nm (FV1000), with emissions collected from 440 to 480 nm, 505 to 550 nm, and 580 to 625 nm for blue, green, and red fluorescence, respectively. Signals from individual fluorophores were collected sequentially (i.e., only one excitation line was used at a time). Pinhole size was adjusted automatically by the software to the optimum for the excitation and emission wavelengths used to image anti-Kcne2 immunoreactivity. Axial resolution (focal width half maximum) was <1 µm for all images acquired. Image postprocessing was restricted to global enhancement of brightness and contrast, as well as cropping, downscaling, and subselecting fluorescent channels, as required, and was performed using ImageJ and Fiji software (National Institutes of Health, Bethesda, MD, USA).^20^

### Data Sharing Statement

Data presented in this work will be made available upon request.

## Results

**Figure 1.**
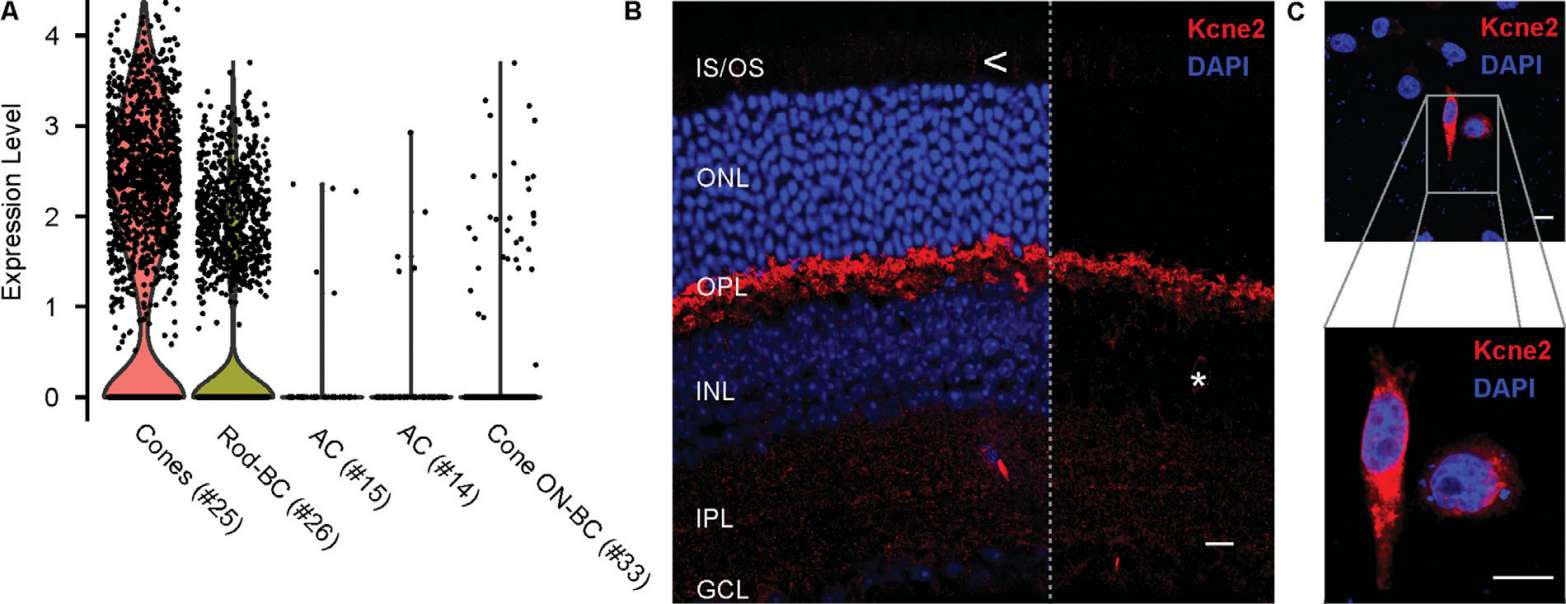
Kcne2 expression in the mouse retina. (**A**) Expression levels of *Kcne2* in single retinal cells as obtained from an analysis of the transcriptome-aligned raw data published by Macosko et al.^5^ The scatter and violin plot shows only cells belonging to the five cell clusters with the highest average expression levels of *Kcne2*. The numbers in brackets indicate cluster numbers as given in Macosko et al.^5^ The rationale for assigning clusters 26 and 33 to the respective BC subtypes is given in Supplementary Figure S1. (**B**) Immunohistochemistry performed on cross-sections from mouse retina showing Kcne2 immunoreactivity (red) is largely restricted to the outer portion of the OPL. Very low levels of immunoreactivity can also be observed the IS/OS layer (highlighted by <) and in sparse cells in the INL (highlighted by *); for details, see Figures 3 and 4, respectively. In the right portion of the image, the blue channel (4′,6-diamidino-2-phenylindol) has been removed to highlight the pattern of Kcne2 immunostaining. Note that the single spot of high-intensity Kcne2 immunoreactivity in the IPL was only observed on single occasions. For details, see Supplementary Figure S2. (**C**) Chinese hamster ovary cells sparsely transfected with a plasmid encoding *Kcne2* showed anti-Kcne2 immunofluorescence only in a subset of cells, indicating sensitivity of the employed antibody. Expression levels as given in (**A**) are gene counts after regression of technical variance in the dataset. GCL, ganglion cell layer; IPL, inner plexiform layer; ONL, outer nuclear layer. *Scale bar*: 10 µm.

For an in-depth analysis of the expression and localization of *Kcne2* in the retina, we first examined the transcriptome-aligned raw data from a published and widely validated single-cell RNA-sequencing dataset.^5^^,^^21^ In accordance with the original observation by Macosko et al.,^5^ *Kcne2* expression was highest in the cluster representing cone photoreceptors (being detected in 71.6% of the cells in this cluster). Notably, high levels of *Kcne2* were also observed in a subpopulation of bipolar cells (BCs; clusters 26 and 33, according to Macosko et al.,^5^ detected in 34.0% and 4.6% of all cells in the respective cluster) and amacrine cells (ACs; clusters 15 and 14, according to Macosko et al.,^5^ detected in 8.8% and 5.3% of all cells, respectively) (Fig. 1A). Note that the low detection frequencies reported for *Kcne2* in clusters 33, 15, and 14 will (at least partially) reflect the reduced detection probability for genes expressed at a low level in the sequencing reaction of the data analyzed here. A summary of the expression levels of Kcne2 in all retinal cell types is given in Supplementary Figure S1B. To explore further which of the BC subpopulations express *Kcne2*, we identified the marker genes that distinguish BC clusters 26 and 33. As shown in Supplementary Figure S1A, the rod-BC marker *Prkca*,^22^ encoding protein kinase C alpha (PKCα), and *Pcp2*^23^ mark cluster 26. Cluster 33 in particular is characterized by the presence of the cone-BC marker *Scgn*^24^ and the ON-BC markers *Vsx1 and* *Grm6*,^25^ suggesting that it represents a subtype of cone ON-BCs, most likely type 6 or 7 (see Supplementary Table S2 in Ref. 26).

Following the bioinformatics analysis of expression data, we moved on to characterize the localization of Kcne2 in the retina by immunohistochmistry. We began by confirming that the candidate antibody raised against the rat sequence of Kcne2 was able to recognize mouse Kcne2. In Chinese hamster ovary cells transfected with a plasmid carrying mouse *Kcne2*, strong Kcne2 immunoreactivity was observed in a portion of the cells (Fig. 1C), whereas no such immunoreactivity could be seen in untransfected controls (not shown). Subsequent immunostaining of mouse retinal cross-sections revealed strong Kcne2 immunoreactivity in the outer portion of the outer plexiform layer (OPL) (Fig. 1B). In addition, a faint signal was obtained from the inner segment (IS)/outer segment (OS) layer, as well as around single cell somata located in the inner nuclear layer (INL) (Fig. 1B, Supplementary figure S2).

**Figure 2.**
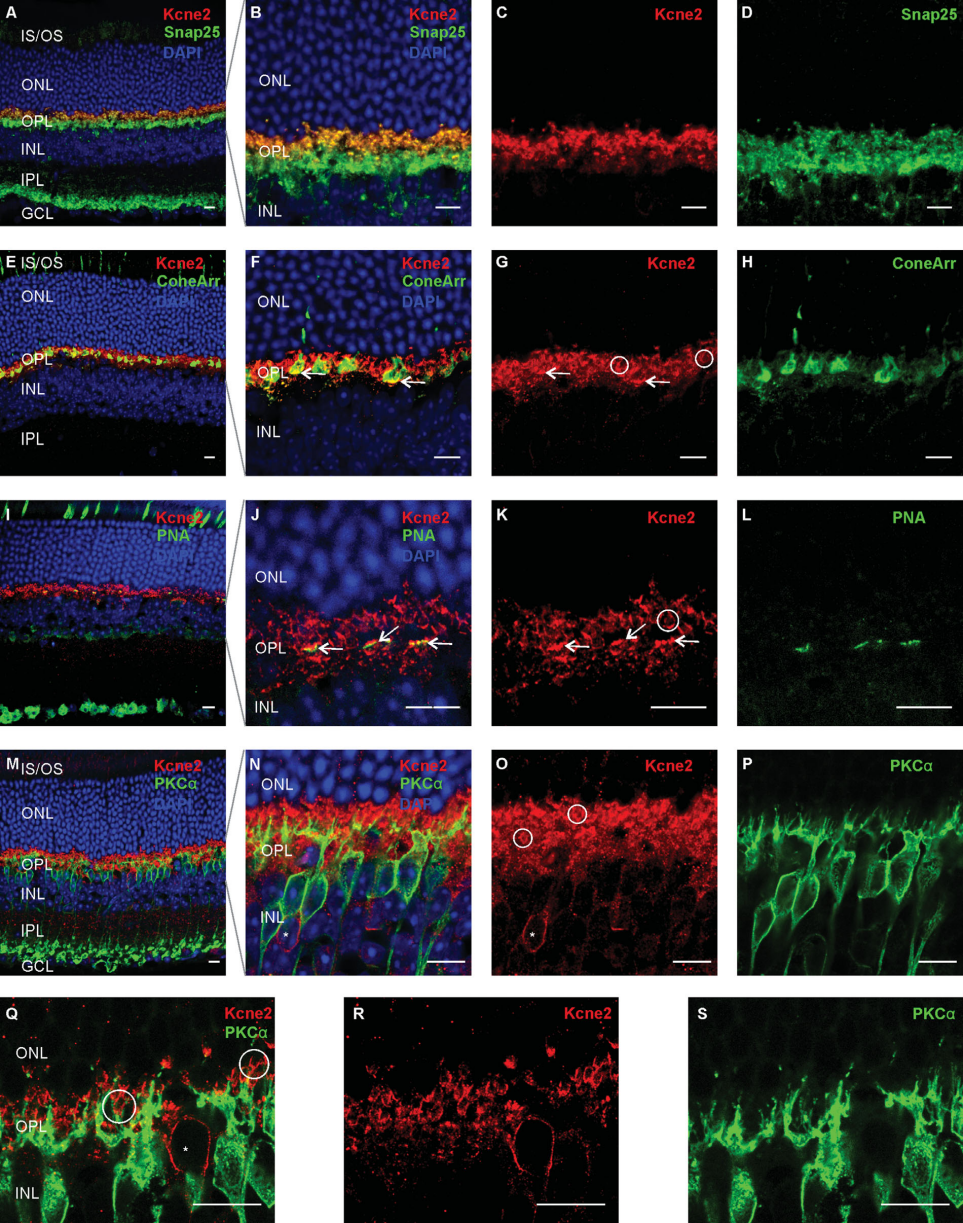
Kcne2 expression in the OPL of the mouse retina. Cross-sections from mouse retina double labeled with Kcne2 (red) and the presynaptic marker Snap25 (**A–D**, green), the cone marker cone-arrestin (**E–H**, green), peanut agglutinin labeling cone pedicles (**I–L**, green), and the rod bipolar cell (rod-BC) marker PKCα (**M–S**, green). Overview images of the entire retinal cross-section are shown in (**A)**, (**E)**, (**I)**, and (**M)**. Data show that Kcne2 co-localizes with Snap25 in the outer portion of the OPL (**A–D**). A portion of this Kcne2 immunoreactivity was localized at the distal surface of the cone pedicles (**E–L**, white arrows). In addition U-shaped Kcne2-immunopositive structures were observed (white circles) that showed spatial association with dendritic terminals of rod-BCs (**M–S**). A subset of bipolar cells with somata that are positive for Kcne2 but negative for PKCα (**N**, **O**, **Q**, **R**, asterisks) can also be observed. Note that immunostaining against PKCα was performed with two different antibodies, one risen in rabbit (**M**–**P**) and one risen in mouse (**Q**–**S**). GCL, ganglion cell layer; IPL, inner plexiform layer. *Scale bar*: 10 µm.

To study further the location of Kcne2 in the OPL in particular, we performed double labeling of Kcne2 in combination with a set of established structural markers. Double labeling for the synapse marker Snap25 revealed that Kcne2 localizes within the synaptic structures in this layer (Figs. 2A–2D). Subsequent double labeling with the cone marker arrestin-3 (cone arrestin) and peanut agglutinin revealed that a part of the Kcne2 immunoreactive structures—namely, those with a rather horizontal, band-like appearance—were cone synapses (Figs. 2E–2L, arrows). Our bioinformatic analysis revealed that, besides cones, *Kcne2* expression was highest in rod-BCs. Therefore, we hypothesized that the remaining, frequently U-shaped, non-cone Kcne2 immunoreactivity at the level of the OPL might represent the afferent synapse of the rod-BCs (Fig. 2, circles). Indeed, double labeling with the rod-BC-marker PKCα showed overlap of both signals at these U-shaped structures, but not for the soma of PKCα-positive rod-BCs (Figs. 2M–2S). Occasionally an extensive overlap along the “U” could be observed (Fig. 2Q, left circle). However, more frequently the Kcne2 immunopositive “U” was met by PKCα-positive structures at its base (Fig. 2Q, right circle).

**Figure 3.**
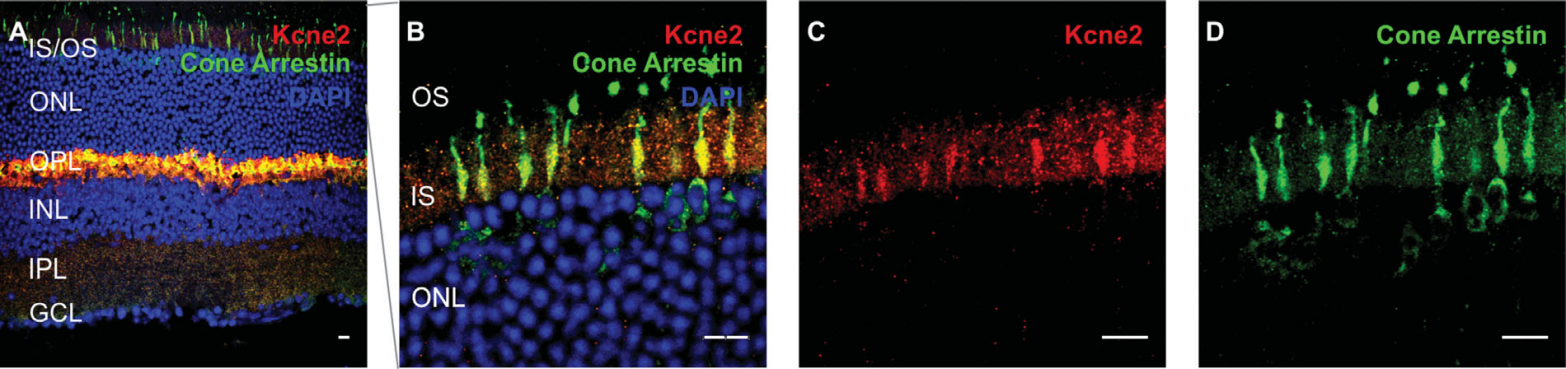
Kcne2 expression in cone inner segments of the mouse retina. Cross-sections from mouse retinae double labeled with Kcne2 (red) and the cone marker cone arrestin (**A**–**D**, green). Overview images of the entire retinal cross-section are shown in the left column (**A**). Kcne2 immunoreactivity was seen in cone photoreceptor inner segments. Rod inner segments exhibited an unspecific fluorescence mostly not above levels of fluorescence commonly observed in this layer plus some punctate signal unrelated to any anatomical structure, conceivably minor unspecific binding of the antibody employed. GCL, ganglion cell layer; IPL, inner plexiform layer. *Scale bar*: 10 µm.

**Figure 4.**
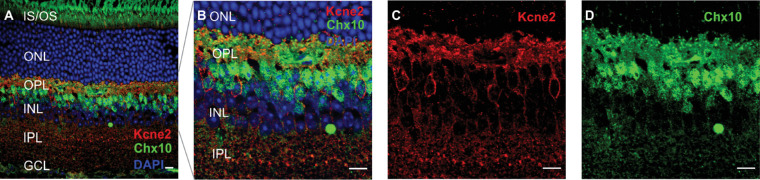
Kcne2 immunoreactivity in the soma of cone ON-BCs. Cross-sections from mouse retinae double labeled with Kcne2 (red) and the cone marker Chx10 (**A**–**D**, green). Cells immunopositive for Kcne2 in the membrane around the soma are primarily located at the outer portion of the INL and are also weakly positive for Chx10. GCL, ganglion cell layer; IPL, inner plexiform layer. *Scale bar*: 10 µm.

As previously mentioned, Kcne2 immunoreactivity was strongest in the OPL but could also be observed at the IS/OS layer and the INL. In the IS/OS layer, Kcne2 signal was observed in single inner segments. Double labeling with the cone markers cone arrestin (Figs. 3A–3D) and peanut agglutinin (Supplementary Fig. S3) revealed that these segments belonged to cone photoreceptors. Note that there was some punctate fluorescence seen outside the cone IS, which was sparse and did not correlate to any anatomical structure. Hence, this is most likely attributable to some weak unspecific binding of the Kcne2 antibody that we used. At the level of the INL, we initially assumed that the sparse cells exhibiting somatic immunoreactivity for Kcne2 may represent a subset of rod-BCs. However, there was no overlap between PKCα- and Kcne2-immunoreactive cells (Figs. 2I–2L). Rather, Kcne2 immunoreactivity overlapped with cells that were weakly positive for the pan-BC marker Chx10 (*Vsx2*). Such weak Chx10 signals would be expected for type 6 or 7 BCs (Figs. 4A–4D).^26^ Notably, these Kcne2 immunoreactive cells also had morphologies resembling BCs. To exclude any doubt that these cells might actually represent amacrine cells, we performed double labeling with a number of classical amacrine cell markers including glycine transporter-1 for glycinergic amacrine cells, gamma aminobutyric acid (GABA) for GABAergic amacrine cells, and choline acetyltransferase for starburst amacrine cells, which showed no overlap with Kcne2-immunoreactive cells (Supplementary Fig. S4). Together with the retinal single cell gene expression data, this suggests that Kcne2 expression in the INL is restricted to cone ON-BCs.

**Figure 5.**
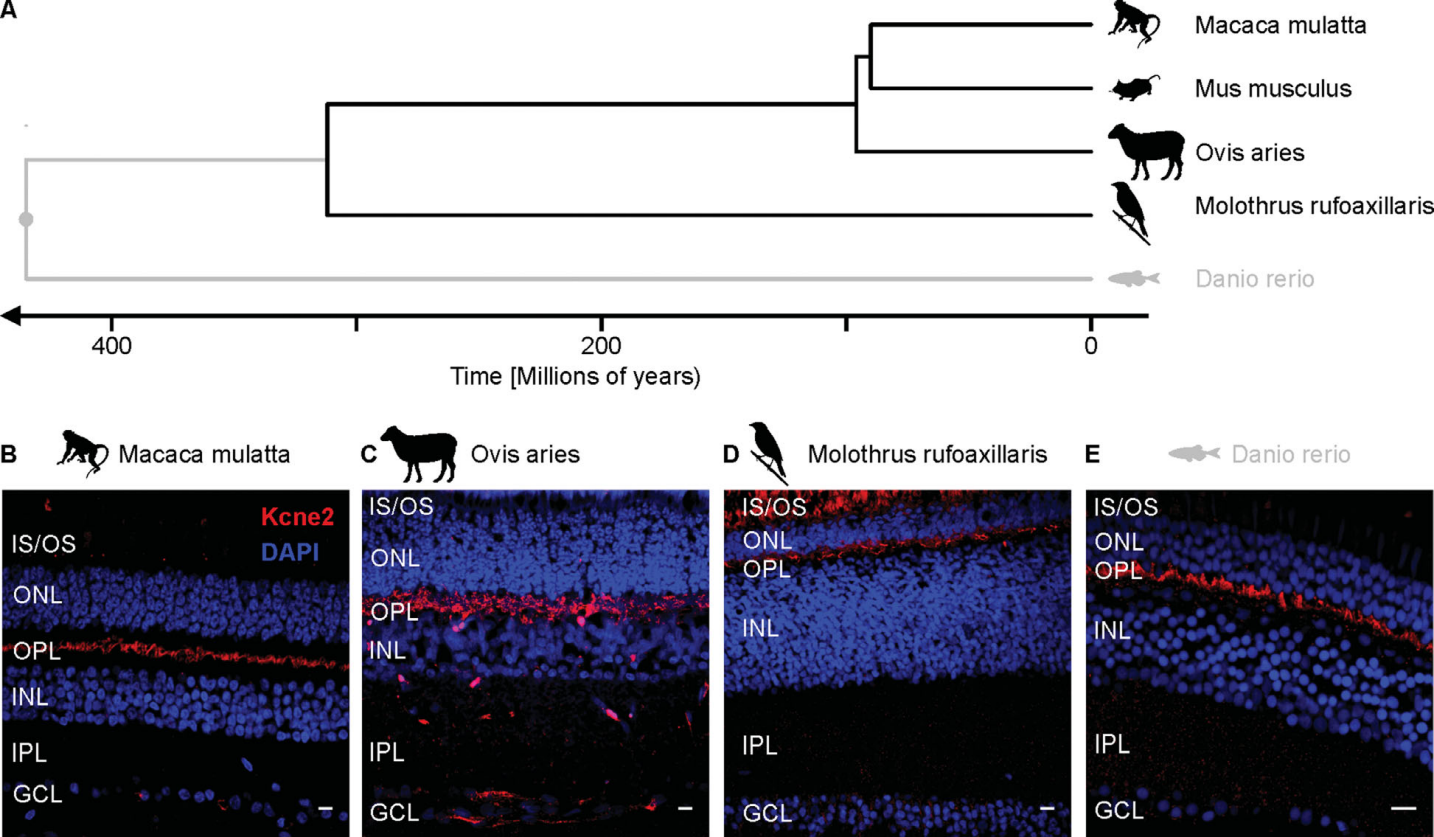
Kcne2 expression in the OPL is conserved among vertebrates. (**A**) Phylogenetic tree for the five vertebrate species included in this study spanning 435 million years of evolution. Note that the zebrafish (*Danio* rerio) is shown in gray to highlight the relatively low sequence homology of its closest relative to Kcne2 (Kcne2-like protein) to the antigen epitope used to raise the Kcne2 antibody. (**B–E**) Immunohistochemistry performed on retinal cross-sections consistently shows immunoreactivity for Kcne2 (red) in the OPL of all studied species. In the Argentinian cowbird (*Molothrus rufoaxillaris*), immunoreactivity can also be observed in the IS/OS layer. Note that data from the mouse are shown in Figure 1. GCL, ganglion cell layer; IPL, inner plexiform layer. *Scale bar*: 10 µm.

Given this defined pattern of Kcne2 immunoreactivity in the mouse retina, we went on to examine to what extent this observation could be generalized to other vertebrate species. In addition to mouse (*Mus musculus*), we examined tissue from macaque (*Macaca mulata*), sheep (*Ovis aries*), Argentinian cowbird (*Moluthrus rufiaxillaris*), and zebrafish (*Danio* rerio). Together, these species span 435 million years of evolution (Fig. 5A). Immunostaining for Kcne2 of retinal cross-sections from all of these species revealed high signal intensities in the OPL. Notably, in none of these species did we observe a signal arising from somata in the INL, as we did in mice. Also, the signal from the IS/OS layer was absent in most species, except for cowbird, where the immunoreactivity in this layer was more pronounced (Figs. 5B–5E). Although these data suggest that Kcne2 localization to the OPL is a highly conserved retinal feature among the entire vertebrate kingdom, it is worth mentioning that only mouse, macaque, sheep, and cowbird have *Kcne2* orthologs. In zebrafish, a *Kcne2* ortholog has not been identified. As expected, mapping the epitope sequence of the Kcne2 antibody employed in this study against the *Kcne2* transcripts (and the predicted Kcne-like transcripts for zebrafish [XP_017213386.1]) of each of these species revealed high sequence homology for all except zebrafish (Supplementary Fig. S1C). For zebrafish, we mapped the epitope sequence against all predicted coding transcripts and did not find any other gene that would encode a protein with a high degree of homology with this epitope (data not shown). Together, the present data indicate that Kcne2 localization in the OPL is conserved over at least 300 million years (as long as a *Kcne2* homolog for zebrafish has not been found) and potentially 435 million years.

## Discussion

The present study provides an in-depth characterization of the expression and localization patterns of Kcne2 in the vertebrate retina. It demonstrates that Kcne2 predominantly localizes to the OPL, where it appears distinctly at the first synapse in the visual pathway in cones and presumably rod-BCs. We found this pattern of expression not only in mice but also in other species spanning 300 million years of evolution. It is thereby likely to be conserved throughout the vertebrate kingdom. In addition, our data show that in the mouse retina, Kcne2 is present in the cone photoreceptor inner segments, as well as in a subset of cone ON-BCs (likely representing type 6 or 7 BCs).

It is worthwhile highlighting the fact that the localization of Kcne2 in the OPL is also conserved in primates. It is therefore highly likely that this is the case in humans, as well, underpinning the translatability of our findings. From a clinical perspective, knowledge on Kcne2 in the OPL might be important for a better understanding of the pathophysiology of (electronegative) retinal dystrophies or autoimmune phenomena such as melanoma-associated retinopathy. Even more important, our data also indicate that patients suffering from Kcne2-associated LQT6^3^ should undergo ophthalmologic examinations to unravel a potential impairment of the retinal function. Should future studies indeed reveal that *Kcne2* mutations cause retinal dystrophy (or dysgenesis), patients diagnosed with LQT6 should routinely be seen by an ophthalmologist. Vice versa, for patients initially diagnosed with a retinal phenotype associated with Kcne2 mutations, cardiologic assessment should be considered to anticipate the time LQT6 is diagnosed. This might potentially prevent the occurrence of lethal arrhythmias as a primary manifestation of LQT6.

Our findings are consistent with the preliminary literature available regarding Kcne2 expression in the retina. At the transcriptional level, Macosko et al.^5^ identified *Kcne2* as an mRNA marker for cones, and, in another work from the same authors, expression of *Kcne2* in rod-BCs has also been reported.^26^ Notably, neither report provides detailed data on Kcne2 protein localization in the retina. To the best of our knowledge, the only description of Kcne2 protein in the eye is provided in a paper published by Zhang et al.,^4^ where, consistent with our data, the authors commented that Kcne2 immunoreactivity can be observed in the OPL of the bovine retina. They did not, however, show any data, as this observation was outside the scope of their study.

Kcne2 expression in the retina is hardly surprising given that it has been found in a variety of tissues, and is frequently referred to as being “ubiquitous.”^1^ However, the characteristic, and well-conserved pattern of Kcne2 localization found in this study would indicate a highly specific role for *Kcne2* in retinal function. We could attribute the majority of the Kcne2 signal to the terminal of the photoreceptor-BC synapse in cones and likely rod-BCs. Kcne2 thereby localizes into an extremely specialized, speed-optimized synapse with an extensively researched but nevertheless incompletely understood signaling machinery.^6^^,^^7^

Although our data firmly indicate that Kcne2 localizes into cone terminals, the evidence that Kcne2 is also present in rod-BC dendrites leaves some room for discussion: Our RNA-sequencing analysis shows expression of *Kcne2* in rod-BCs on mRNA level. This finding is in line with reports from others, who observed increased expression levels of Kcne2 by data from cell-sorted rod-BC bulk RNA-sequencing or single-cell RNA-sequencing.^26^^–^^28^ However, we did not observe a 1:1 overlap of immunoreactivity for Kcne2 and the rod-BC marker PKCα. Rather, Kcne2 immunoreactivity was arranged in U-shaped patterns remotely from the rod-BC somata where PKCα staining is becoming faint. In some cases, there seemed to be indeed a wide overlap of PKCα and Kcne2 immunoreactivity; in others, PKCα-immunoreactive rod-BC dendrites only joined those U-shaped structures at their base (Fig. 2Q, circles). Notably, for proteins clearly established to be localized in rod-BC dendritic terminals, such as Trpm1, immunoreactivity has also been observed to extend beyond the PKCα-positive parts of rod-BCs (e.g., Morgans et al.^29^). The pattern of immunoreactivity for Kcne2 we see is, therefore, in keeping with Kcne2 localizing into rod-BC dendritic terminals. Still, localization of Kcne2 in rod spherules or horizontal cell synapses should be considered as alternative explanations for our observations. Rod spherules do demark as U-shaped complexes (e.g., in ribeye^30^ or PSD95^31^ immunostaining); yet, these usually have their base toward the outer nuclear layer (ONL), which is inverse to what we observed for Kcne2. Horizontal cell neurites are predominantly found in the inner part of the OPL,^31^ which is also distinct from where we observed Kcne2 immunoreactivity. With this and the RNA-sequencing results in mind, we think that the none-cone portion of the OPL Kcne2 immunoreactivity indeed arises from rod-BCs. However, further studies, such as with immunogold electron microscopy, are required to confirm our interpretation.

In the rod-BC postsynaptic compartment, fast metabotropic transduction of visual signals propagated from the photoreceptors is enabled by a multi-protein complex that encompasses the glutamate receptor mGluR6, the effector channel Trpm1, and several auxiliary proteins and co-receptors.^32^ The six transmembrane-domain potassium channels that act as the classical interaction partners of β-subunits of the Kcne family have as yet not been found to form part of this particular complex. However, K_v_1.2, K_v_1.3, and the hyperpolarization-activated cyclic nucleotide-gated (HCN) channel Hcn2 are all reportedly expressed in rod-BC dendrites and are known to interact with Kcne2.^2^^,^^33^^–^^37^ In cone synaptic terminals, voltage-gated calcium channels (Cav1.4; again as part of a multi-protein signaling complex) are essential for mediating calcium influx and thereby neurotransmitter release. Also, cyclic nucleotide-gated and HCN channels have been identified in cone synapses.^33^^,^^35^ Remarkably, Hcn3 specifically localizes to cone pedicles. As Hcn channels were shown to interact with Kcne2, this would be an interesting candidate interaction partner for further investigation.^35^ Ongoing research may be able to determine if Kcne2 at this synapse interacts with already known or novel interaction partners and what the functional consequences of such interactions could be.

In mice, we observed Kcne2 immunoreactivity in cone inner segments. Here, several ion channels that are well known to interact with Kcne2 are localized, including K_v_2.1, Hcn1, and possibly also K_v_11- and K_v_7-family channels.^33^ It is particularly exciting to speculate on a potential interaction of Kcne2 and K_v_2.1 within cones: K_v_2.1 forms heteromers with another Kv-subunit, K_v_8.2, in both cones and rods. Loss-of-function mutations in K_v_8.2 cause a hereditary retinal disorder known as cone dystrophy with supernormal rod response (CDSRR), most likely by increasing potassium conductance (I_k,x_).^38^^,^^39^ Interestingly, although K_v_8.2 and K_v_2.1 are expressed in both rods and cones, CDSRR is characterized by a selective early-onset degeneration of cones, whereas rods are more resilient. It is conceivable that in cones the expression of Kcne2 modulates K_v_2.1 in a way that the absence of functional Kv8.2 in CDSRR has an even higher impact on I_k,x_ and thereby promotes cone susceptibility.

Although we could show robustly that Kcne2 expression in the cone and rod-BC synapses contributes to the Kcne2 immunoreactivity observed in the outer portion of the OPL, we did not prove that this immunoreactivity is exclusively due to Kcne2 expression in these two cell types. From the present data we cannot exclude the possibility that Kcne2 also localizes to the synapse in type 6 and 7 cone ON-BCs (where we detected *Kcne2* on the transcriptome level). As yet, however, there is no evidence for Kcne2 expression in any other cells that form synapses in the OPL. It is also worth mentioning that we were not able to identify Kcne2 staining in any class of amacrine cell, although we found two clusters that showed low *Kcne2* expression in the single-cell RNA-sequencing data. One possible explanation for this is that single-cell RNA-sequencing data were obtained from 14-day-old mice, an age at which retinal development is not fully complete, whereas the histology was performed on adult mice 4 to 6 weeks old. Thus, Kcne2 expression in amacrine cells may only be present during development and is absent in the adult.

In summary, we have shown that in the retina Kcne2 exhibits a distinct, well-defined expression pattern predominantly localizing in the OPL at cone photoreceptor and probably rod bipolar cell synapses and is conserved through the vertebrate kingdom. Although the functional role of Kcne2 in the retina remains to be studied, the data reported herein offer new potential avenues toward a better understanding of cone phototransduction as well as the formation of the highly specialized photoreceptor–bipolar cell synapse.

## Supplementary Material

Supplement 1

Supplement 2

Supplement 3

Supplement 4
