# Supplementary material for: Expression and Localization of Kcne2 in the Vertebrate Retina
Source: Invest Ophthalmol Vis Sci. 2020 Mar 19;61(3):33. doi: 10.1167/iovs.61.3.33 (PMC7401445; doi:10.1167/iovs.61.3.33)
Supplement: Supplement 1 [file iovs-61-3-33_s001.pdf]

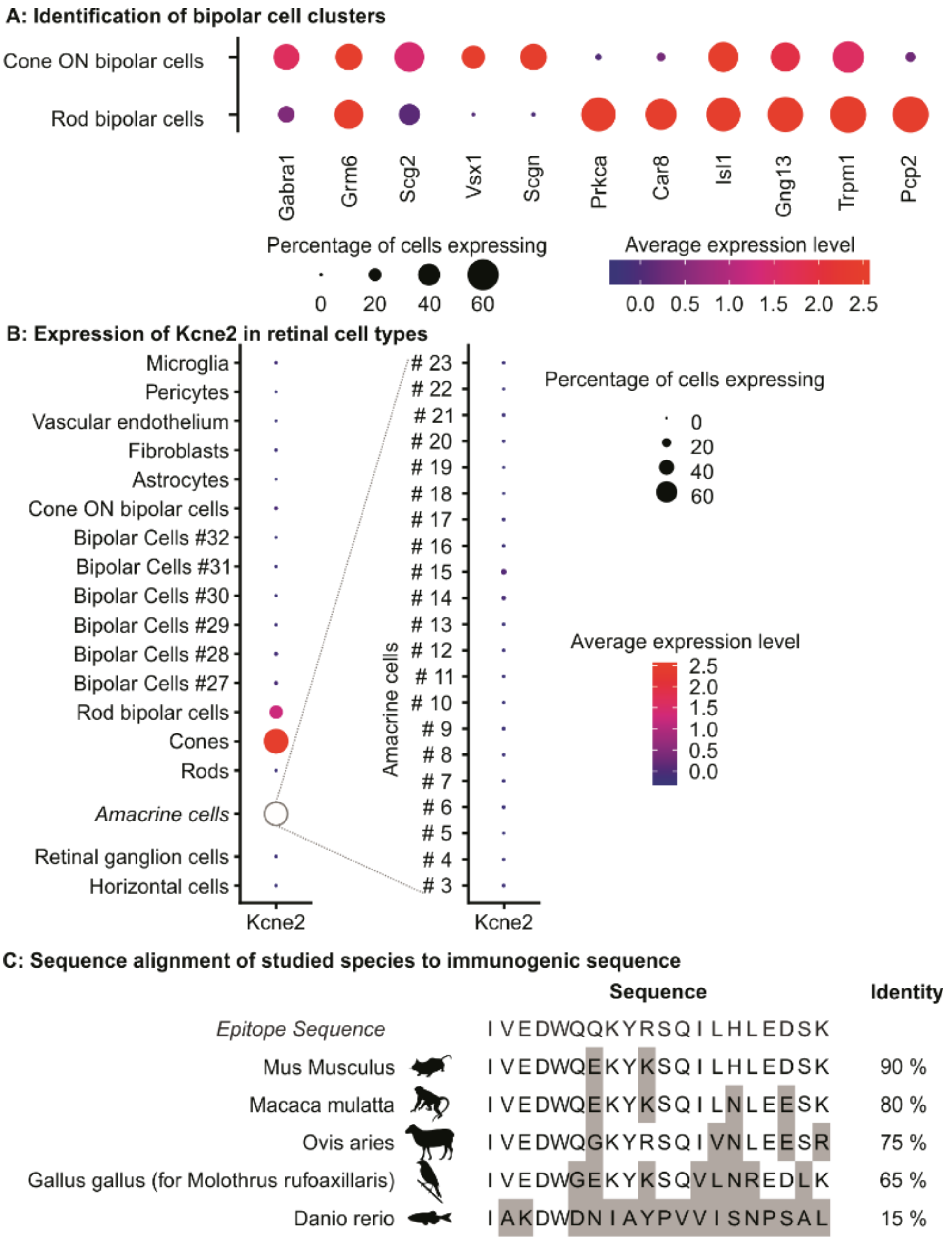

Suppl. Figure 1: Assignment of biological bipolar cell-subtypes to the bipolar cell clusters with the highest expression levels of *Kcne2* (A), expression levels for Kcne2 in each cluster (B) and alignment of the immunogenic sequence for the Kcne2 antibody used in this study to the sequences of the Kcne2 orthologues (respectively homologues for Zebrafish [Danio rerio], C). A and B) Dot plots showing the average expression level (circle colour) and the percentage of cells where the gene was encountered (circle size) for bipolar cell marker genes (A) and *Kcne2* (B). A) Cluster #26 from Macosko et al. was identified to represent rod bipolar cells (BCs) by selective expression of Protein kinase C α (Prkca) and Purkinje cell protein 2

(Pcp2).Cluster #33 was considered to represent a subset of cone ON-bipolar cells (presumably Type 6 or 7 BCs) as for the detection of cone-BC and ON-BC markers (e.g. Scgn, Vsx1, Grm6). C) Sequence homology between the antigen epitope for the Kcne2 antibody and the respective Kcne2 orthologues was high for all species except for zebrafish.
