# Supplementary material for: Expression and Localization of Kcne2 in the Vertebrate Retina
Source: Invest Ophthalmol Vis Sci. 2020 Mar 19;61(3):33. doi: 10.1167/iovs.61.3.33 (PMC7401445; doi:10.1167/iovs.61.3.33)
Supplement: Supplement 2 [file iovs-61-3-33_s002.pdf]

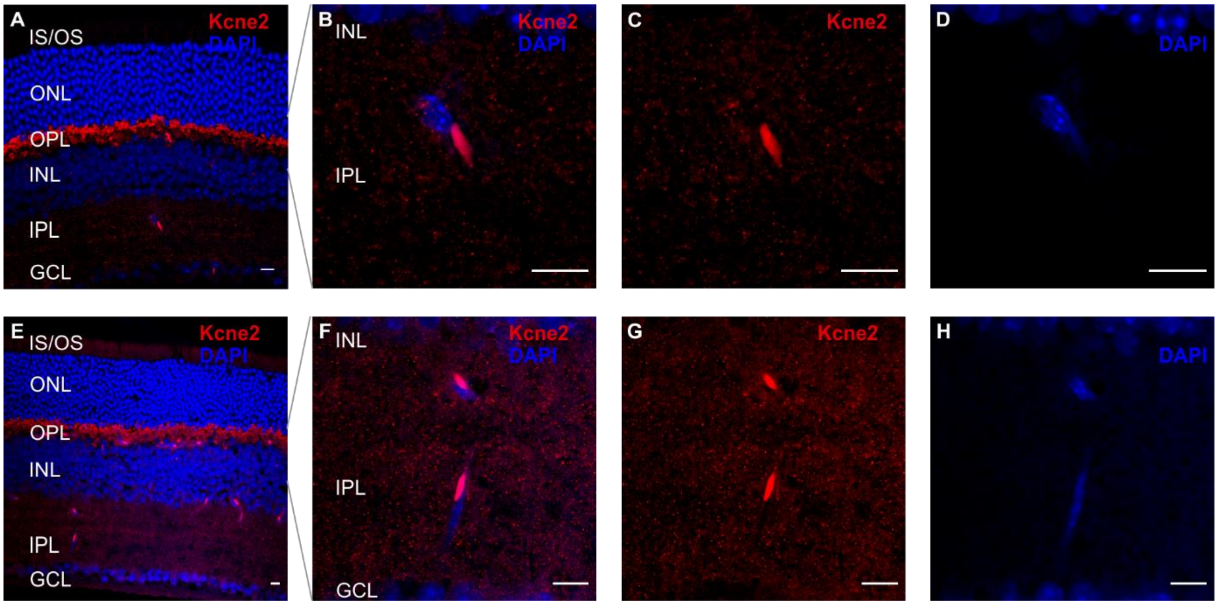

Suppl. Figure 2: Kcne2 immunostaining in the inner plexiform layer. Cross-sections from mouse retinae labelled with Kcne2 (red) and counterstained with DAPI (blue, A-H). Both stainings were performed at the same day on tissue obtained from the same eye. Only in this round of staining spots of increased Kcne2 immunoreactivity were observed in the IPL which we further analysed to evaluate the possibility that these might represent displaced amacrine cells. Indeed we found that these spots were adjacent to areas of typical nuclear DAPI staining, however they seemed to be not associated to any distinguishable subcellular structure. Rather, on closer contemplation it became apparent that the signal in the red (Kcne2) channel was directly matched by signal in the blue (DAPI) channel. Together this indicates that the presumed Kcne2 immunoreactivity in the IPL does not arise from displaced amacrine cells. Rather, these might be blood cells with a high nucleus/soma ratio.. A is a repeat of **Figure 1** B. GCL: Ganglion cell layer, IPL: Inner plexiform layer, INL: inner nuclear layer, OPL: outer plexiform layer, ONL: Outer nuclear layer, Inner and outer segment layers: (IS, OS). Scale bar: 10 µm.
