# Supplementary material for: Expression and Localization of Kcne2 in the Vertebrate Retina
Source: Invest Ophthalmol Vis Sci. 2020 Mar 19;61(3):33. doi: 10.1167/iovs.61.3.33 (PMC7401445; doi:10.1167/iovs.61.3.33)
Supplement: Supplement 3 [file iovs-61-3-33_s003.pdf]

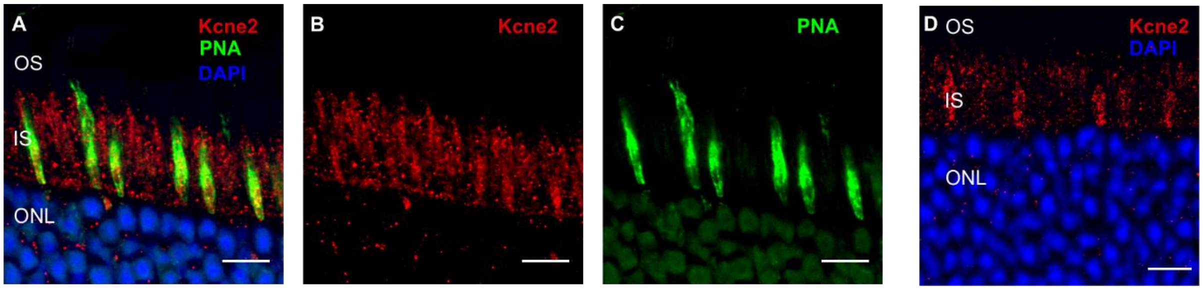

Suppl. Figure 3: Kcne2 expression in cone inner segments of the mouse retina. A – C) Cross-section from mouse retina double-labelled with Kcne2 (red) and the cone marker peanut agglutinin (PNA). D) Cross-section from mouse retina labelled with anti-Kcne2 antibody only. Kcne2 immunoreactivity was seen in cone photoreceptor inner segments, ONL: Outer nuclear layer, Inner and outer segment layers: (IS, OS). ONL: Outer nuclear layer, OS: Outer segments. See Figure 1 for labelling of the individual retinal layers in overview images (A, E, I) Scale bar: 10 µm.
