# Supplementary material for: Expression and Localization of Kcne2 in the Vertebrate Retina
Source: Invest Ophthalmol Vis Sci. 2020 Mar 19;61(3):33. doi: 10.1167/iovs.61.3.33 (PMC7401445; doi:10.1167/iovs.61.3.33)
Supplement: Supplement 4 [file iovs-61-3-33_s004.pdf]

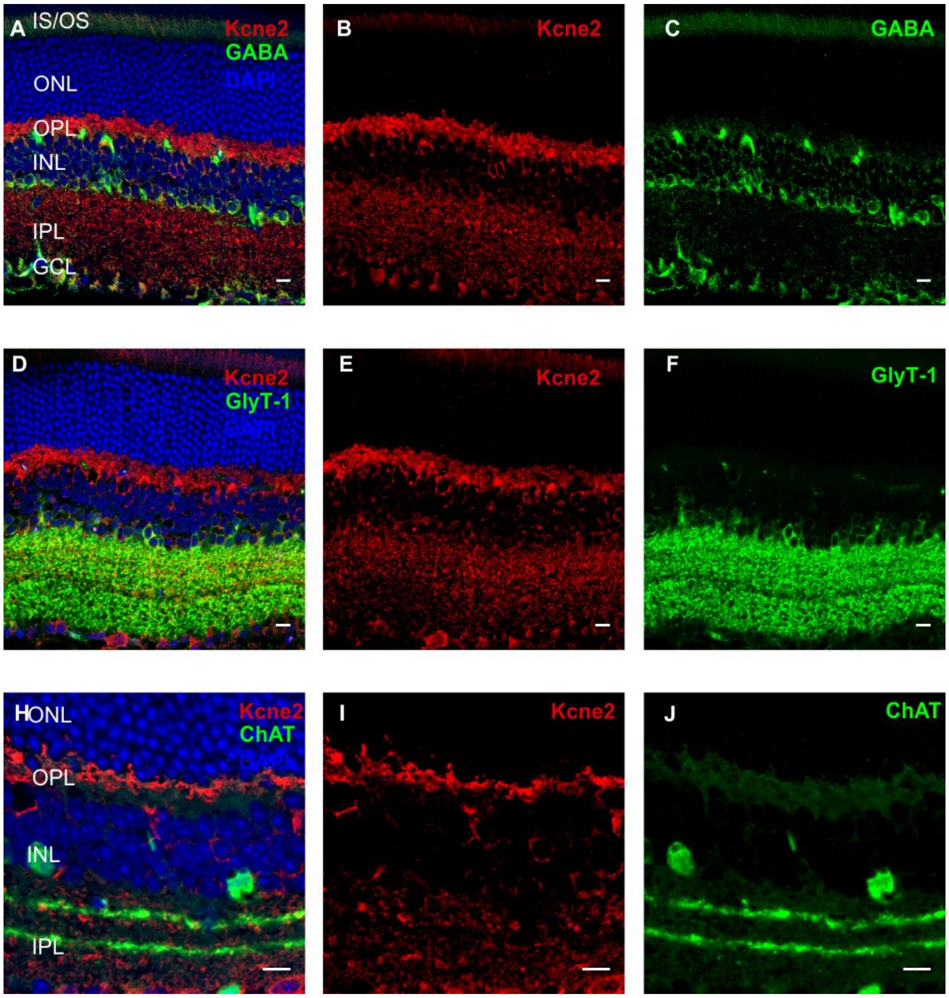

Suppl. Figure 4: Cross-sections from mouse retinae double-labelled with Kcne2 (red) and markers for different subtypes of amacrine cells (all green). A-C) GABA, D-F) GlyT-1, G-I) ChAT. Cells with Kcne2 positive somata were not positive for any of these markers. GCL: Ganglion cell layer, IPL: Inner plexiform layer, INL: inner nuclear layer, OPL: outer plexiform layer, ONL: Outer nuclear layer, Inner and outer segment layers: (IS/OS). Scale bar: 10 µm.
